# Classification and Authentication of Paprika by UHPLC-HRMS Fingerprinting and Multivariate Calibration Methods (PCA and PLS-DA)

**DOI:** 10.3390/foods9040486

**Published:** 2020-04-13

**Authors:** Sergio Barbosa, Javier Saurina, Lluís Puignou, Oscar Núñez

**Affiliations:** 1 Department of Chemical Engineering and Analytical Chemistry, University of Barcelona, Martí i Franquès 1-11, E08028 Barcelona, Spain; sergiobarbosabarbero@hotmail.com (S.B.); xavi.saurina@ub.edu (J.S.); lluis.puignou@ub.edu (L.P.); 2 Research Institute in Food Nutrition and Food Safety, University of Barcelona, Recinte Torribera, Av. Prat de la Riba 171, Edifici de Recerca (Gaudí), Santa Coloma de Gramenet, E08921 Barcelona, Spain; 3 Serra Húnter Fellow, Generalitat de Catalunya, Rambla de Catalunya 19-21, E08007 Barcelona, Spain

**Keywords:** UHPLC-HRMS fingerprinting, non-targeted analysis, food authentication, paprika, product designation of origin, principal component analysis, partial least squares regression-discriminant analysis

## Abstract

In this study, the feasibility of non-targeted UHPLC-HRMS fingerprints as chemical descriptors to address the classification and authentication of paprika samples was evaluated. Non-targeted UHPLC-HRMS fingerprints were obtained after a simple sample extraction method and C18 reversed-phase separation. Fingerprinting data based on signal intensities as a function of *m/z* values and retention times were registered in negative ion mode using a q-Orbitrap high-resolution mass analyzer, and the obtained non-targeted UHPLC-HRMS fingerprints subjected to unsupervised principal component analysis (PCA) and supervised partial least squares regression-discriminant analysis (PLS-DA) to study sample discrimination and classification. A total of 105 paprika samples produced in three different regions, La Vera PDO and Murcia PDO, in Spain, and the Czech Republic, and all of them composed of samples of at least two different taste varieties, were analyzed. Non-targeted UHPLC-HRMS fingerprints demonstrated to be excellent sample chemical descriptors to achieve the authentication of paprika production regions with 100% sample classification rates by PLS-DA. Besides, the obtained fingerprints were also able to perfectly discriminate among the different paprika taste varieties in all the studied cases, even in the case of the different La Vera PDO paprika tastes (sweet, bittersweet, and spicy) which are produced in a very small region.

## 1. Introduction

Paprika is a red spice obtained after drying and grinding some varieties of peppers of the genus *Capsicum* belonging to the large family of Solanaceae [1]. The five main cultivated and economically important species of paprika are *Capsicum annuum* L., *C. chinense* Jacq. and *C. frutescens* L., now widely grown throughout Europe, the southern United States, Africa, India and China, and the species *C. baccatum* L. and *C. pubescens* Ruiz & Pav., which are grown predominantly in South America [2,3].

Paprika is often used in many foods such as soup, meat, ice cream, baked goods, and in seasoning blends to add color and taste [4], although it is also used in personal protection sprays, medicine, and cosmetics [5,6,7,8,9].

Today, both consumers and food manufacturers are increasingly concerned about quality standards and as a result, there is a growing demand for food traceability. In this context, the designation of Protected Designation of Origin (PDO) is an effective tool to guarantee the quality and geographical origin of a product. In Europe, there are five Protected Designations of Origin for paprika: Piment d’Espelette (France), Paprika Szeged (Hungary), Paprika Žitava (Slovakia Žitava paprika), Pimentón de La Vera (Cáceres, Extremadura, Spain), and Pimentón de Murcia (Murcia, Spain).

To protect these products, the competent authorities carry out various checks, consisting mainly of inspections of production sites. During these inspections, records, raw materials, production systems, maturation, etc. are checked and various samples are taken to be analyzed by independent laboratories to ensure that the whole process complies with the regulations in force.

However, adulteration in natural products remains a widespread practice that mainly seeks economic benefit, either through increased sales or through reduced production costs. This practice is very difficult to combat, mainly due to the globalization of trade, the complexity of supply chains, the regulatory policy differences between each country and the fact that the main legal responsibility for the safety of marketed products is delegated by default to manufacturers [10]. Besides, the methods used for the adulteration of natural products are increasingly sophisticated, and, in some cases, are specially designed to mislead the methods of analysis applied by the competent authorities, which are limited to routine analyses that do not detect adulterations.

However, the high number of illegal compounds, as well as the different ways of adulterating a natural product, makes it sometimes difficult to identify those compounds that should be analyzed. In this context, non-targeted methods encompass the complexity of modern authentication of natural products [11,12,13,14]. The main focus of this type of strategy is to detect as many compounds as possible below 1.500 Da [15]. For this reason, liquid chromatography coupled with high-resolution mass spectrometry (LC-HRMS), such as TOF and Orbitrap, as well as their hybrid configurations, are the most suitable instruments to carry out this type of strategy, since they present a high sensitivity, have a high-resolution power (over 100,000 FWHM, full width at half maximum) and a very accurate mass/charge ratio determination (<5 ppm), able to distinguish between isobaric compounds. Furthermore, data acquisition in high-resolution full scan mode allows the simultaneous combination of targeted and non-targeted analysis, identification of new compounds, and retrospective data analysis [16]. Another important feature of this analytical strategy is that the authentication of a product can be approached from various perspectives in a single analysis. Depending on the selected approach to solve the problem of interest, the fingerprint allows not only detecting the origin or type of raw material used but also detects unlabeled compounds, unauthorized additives or the use of prohibited technological processes, among others [17].

In this type of analytical strategy, sample preparation must be as simple and non-specific as possible, to avoid losses of compounds, accompanied by loss of information [17]. Normally, the sample is extracted with a hydrophilic organic solvent-water mixture.

In non-targeted methods based on fingerprinting, it is not necessary to identify the compounds that are most relevant to product authentication. This type of strategy is often qualitative and based on a comparison between the samples under study and the authentic reference samples, which are used to build an appropriate database. This database is used to compare the fingerprints of the unknown samples with the fingerprints of the authentic reference samples, thus reducing the time and cost of analysis [12]. However, identification is important when regulatory action must be taken based on the results. In a court of law, the identification of the chemical structure of a primary or secondary marker is an important asset in the judicial process [11], although it should be kept in mind that different biomarkers can be obtained depending on the procedure used. The biomarkers obtained depend largely on the overall experimental approach used, including sample handling, separation, and detection, as well as the specific instrumentation used [18].

Analytical signals can be obtained in positive mode, in negative mode or by data fusion. In many cases, the most important aspect is to detect as many signals as possible, under both positive and negative modes and joining all the information in a single matrix, that is by data fusion, a complex authentication problem could be achieved. Another important feature of HRMS instruments such as q-Orbitrap, is their ability to automatically isolate those signals that are more abundant, to be fragmented for future identification, if deemed appropriate.

Data pre-processing is one of the most relevant points to consider in methods based on fingerprinting. Once the data has been generated, it is important to have software that helps to obtain the working matrix. This matrix is constituted by the retention time, the *m/z* values and the area or signal of each detected peak. In this software, some parameters must be set, such as the mass tolerance for peak alignment, the total intensity threshold, the maximum peak displacement, and the S/N threshold. Sometimes, these parameters eliminate chemical interferences from the matrix, but they must be properly adjusted to prevent discarding signals that may be relevant [18]. Besides, depending on the type of study being conducted, it is necessary to reduce the number of signals to simplify the matrix obtained. In these cases, some actions can be taken such as removing signals that are not detected in a minimum percentage of the samples or remove signals that are not observed in the quality controls, which generally consist of a mixture of equal volumes of all the samples analyzed [18].

Once the data matrix has been obtained, it should be evaluated using multivariate statistical models, whether supervised or unsupervised, that finally allow the authentication of the product under study [13]. However, the great diversity of chemometric models, together with the fact that this type of strategy is in its beginnings in the food field, make that there is not yet a clear consensus as to which chemometric model is most suitable for routine use in quality control.

The main objective of this work was to develop a UHPLC-HRMS (Orbitrap) method for the characterization, classification, and authentication of paprika samples using a non-targeted fingerprint approach. Different samples of La Vera paprika, Murcia paprika, and Czech Republic paprika were analyzed using a simple sample extraction procedure. The hypothesis established in this work is that UHPLC-HRMS fingerprint data, obtained in ESI negative mode, can be considered as a source of potential chemical descriptors to be exploited for the characterization and classification of paprika samples by unsupervised and supervised methods such as principal components analysis (PCA) and partial least squares regression-discriminant analysis (PLS-DA).

## 2. Materials and Methods

### 2.1. Chemicals and Standard Solutions

All the reagents, standards and chemicals employed in the present work were of analytical grade. Water, acetonitrile, and methanol (all of them LC-MS Chromasolv^®^ quality), acetone, and formic acid (98–100%) were purchased from Sigma-Aldrich (Steinheim, Germany). Hydrochloric acid (35%) was obtained from Merck (Seelze, Germany).

### 2.2. Instrumentation

An Accela UHPLC instrument from Thermo Fisher Scientific (San Jose, CA, USA), with a quaternary pump, and an autosampler, was employed for the sample chromatographic analysis. Reversed-phase separation in an Ascentis^®^ Express C18 porous-shell (150 × 2.1 mm, 2.7 µm partially porous particle size) column obtained from Supelco (Bellefonte, PA, USA) under universal gradient elution mode using water (solvent A) and acetonitrile (solvent B), both of them containing 0.1% formic acid, was proposed for obtaining the chromatographic fingerprints. The elution gradient program employed begun with an isocratic elution step at 10% B for 1 min, followed by a linear gradient from 10 to 95% solvent B in 19 min. Then, 95% solvent B was kept for 3 min, and back to initial conditions at 10% solvent B in 1 min. A column re-equilibration time of 6 min at 10% solvent B was employed, giving place to a total chromatographic gradient program of 30 min. The mobile phase flow rate was 300 µL/min. The column was kept at room temperature, and an injection volume of 10 µL (at full-loop mode) was employed for sample analysis.

The UHPLC instrument was coupled to a Q-Exactive Orbitrap HRMS instrument (Thermo Fisher Scientific) by employing a heated electrospray ionization source (H-ESI II). Nitrogen (purity of 99.98%) was employed for the H-ESI sheath, sweep, and auxiliary gases at flow rates of 60, 0, and 10 a.u. (arbitrary units). H-ESI was operated in negative ionization mode by applying a capillary voltage of −2.5 kV. H-ESI vaporizer temperature and capillary instrument temperature were kept at 350 °C and 320 °C, respectively. An S-Lens RF level of 50 V was used. Tuning and calibration of the Orbitrap analyzer were performed every 3 days by using the Thermo Fisher Scientific commercially available calibration solution for that purpose. Full scan HRMS spectra (*m/z* 100–1500) at a mass resolution of 70.000 FWHM (full-width at half maximum, at *m/z* 200) was employed to register the UHPLC-HRMS metabolomics fingerprints, with an automatic gain control (AGC) target (which is the number of ions to fill the instrument C-Trap) of 2.5 × 10^5^, and a maximum injection time of 200 ms.

UHPLC-HRMS system control and data processing were performed using Xcalibur version 3.1 software (Thermo Fisher Scientific).

### 2.3. Samples and Sample Treatment

One hundred and five paprika samples obtained from local markets in Spain and the Czech Republic were analyzed. Samples belong to different PDO and production regions, as well as different taste varieties: 65 samples from La Vera PDO (including 23 sweet, 22 bittersweet, and 20 spicy), 17 samples from Murcia PDO (including 8 sweet, and 9 spicy), and 23 samples from Czech Republic (including 7 smoked-sweet, 8 sweet, and 8 spicy).

Samples were extracted following a previously proposed procedure [1,19]. Briefly, paprika samples (0.3 mg) were extracted with water:acetonitrile 20:80 *v/v* solution (3 mL) by stirring (1 min) with a vortex mixer (Stuart, Sone, UK), and by sonication (15 min) with an ultrasonic bath (2510 Branson, Hampton, NH, USA). Centrifugation was then carried out for 15 min at 4500 rpm (Rotana 460 HR centrifuge, Hettich, Germany). The obtained extract was then filtered with 0.45 µm nylon filters (Whatman, Clifton, NJ, USA) and transferred into 2 mL injection vials, which were kept at −18 °C until the UHPLC-HRMS analysis.

Besides, a quality control (QC) solution, employed for the evaluation of the method reproducibility and to ensure the robustness of the chemometric results, was employed. This QC was prepared by mixing 50 µL of each one of the paprika sample extracts obtained.

To prevent signal tendencies attributed to the sample sequence analysis, all paprika samples were analyzed randomly with the proposed UHPLC-HRMS method. Besides, blanks of acetonitrile and QCs were injected every 10 randomly analyzed samples (representing 12 QC analyses).

### 2.4. Data Analysis

Data matrices for untargeted UHPLC-HRMS analysis were obtained with R software (R Foundation, Vienna, Austria). First, UHPLC-HRMS raw data was submitted to MSConvert software to obtain an Excel file with the profile of peak intensities as a function of *m/z* values and retention times for all the chemical features detected. An absolute intensity threshold peak filter of 10^5^ was employed. PCA and PLS-DA chemometric calculations were made by using SOLO 8.6 chemometric software (Eigenvector Research [20], Manson, WA, USA). The theoretical background of these methods in a detailed way is addressed elsewhere [21]. X-data matrices for PCA and PLS-DA were based on the UHPLC-HRMS metabolomic fingerprints (peak intensities as a function of retention time and *m*/*z* values) obtained in H-ESI (-) mode. The PLS-DA Y-data matrix included the analyzed sample classes. Scatter plots of scores of the principal components (PCs) in PCA and the latent variables (LVs) in PLS-DA were used to study the distribution and classification of samples. The applicability of the proposed PLS-DA models for sample classification was assessed by employing 70% of the analyzed samples as the calibration sets (71 samples), while the remaining 30% of samples were used for prediction and validation (31 samples). Optimal number of LV in PLS-DA was determined by considering the first significant minimum point of the cross-validation (CV) error from a Venetian blind approach.

## 3. Results and Discussion

### 3.1. UHPLC-HRMS Metabolomics Fingerprinting Approach

**Figure 1:**
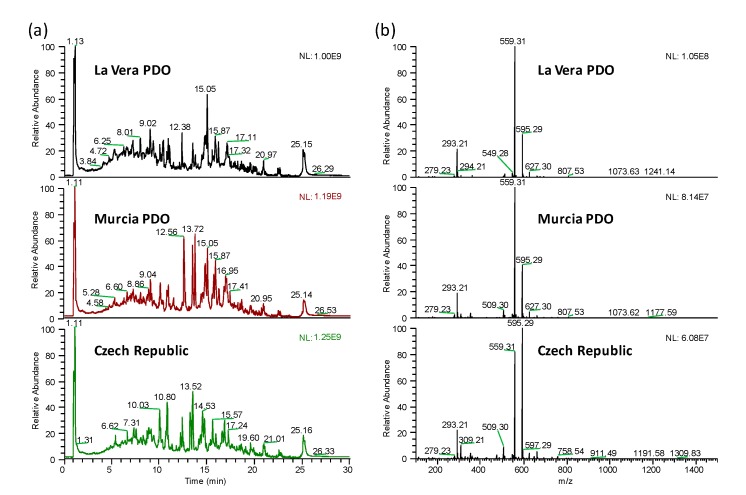
UHPLC-HRMS total ion chromatograms (**a**) and HRMS full scan spectra (*m/z* 100–1.500) for the signal at retention time 15.05 min (**b**) of three selected sweet La Vera PDO, Murcia PDO, and Czech Republic paprika samples.

The main objective of the present work was to evaluate the feasibility of using non-targeted UHPLC-HRMS metabolomic fingerprints as sample chemical descriptors to address the characterization, classification, and authentication of paprika samples by multivariate chemometric methods. For that purpose, 105 paprika samples belonging to different PDO and production regions (La Vera PDO, Murcia PDO, and the Czech Republic), all of them including samples with different taste varieties, were analyzed. A simple and generic sample extraction method using water:acetonitrile 20:80 (*v*/*v*) as extracting solvent was proposed [1,19,22,23]. Reversed-phase UHPLC on a C18 column under universal gradient elution with water and acetonitrile (both of them with 0.1% formic acid) as mobile phase components were selected for the chromatographic separation. As a non-supervised fingerprinting methodology is intended, both sample treatment and chromatographic separation were kept as generic as possible with the aim of extracting as much sample chemical features as possible. Accordingly, an untargeted strategy based on UHPLC-HRMS fingerprints consisting of peak intensities registered as a function of both *m/z* and retention time values was employed. Negative H-ESI ionization mode was selected for the full scan (*m*/*z* 100–1500) data acquisition, taking into consideration that polyphenols and phenolic acids, typically ionized in that way, are among the most common bioactive substances in plant-based products [24]. As an example, Figure 1a shows the total ion chromatograms (TIC) registered in negative ionization mode for three selected sweet paprika samples belonging to different production regions (La Vera PDO, Murcia PDO, and the Czech Republic). Besides, the full scan HRMS corresponding to the signal registered at a retention time of 15.05 min for the same three sweet paprika samples is depicted in Figure 1b.

As can be seen, the proposed methodology provides rich fingerprints for the three types of samples from the point of view of the number of signals detected. However, important differences can be observed among sample types regarding the number of peak signals and their abundances, especially in the range from 5 to 20 min, in both the total ion chromatograms and the HRMS spectra.

### 3.2. Sample Exploration by PCA

**Figure 2:**
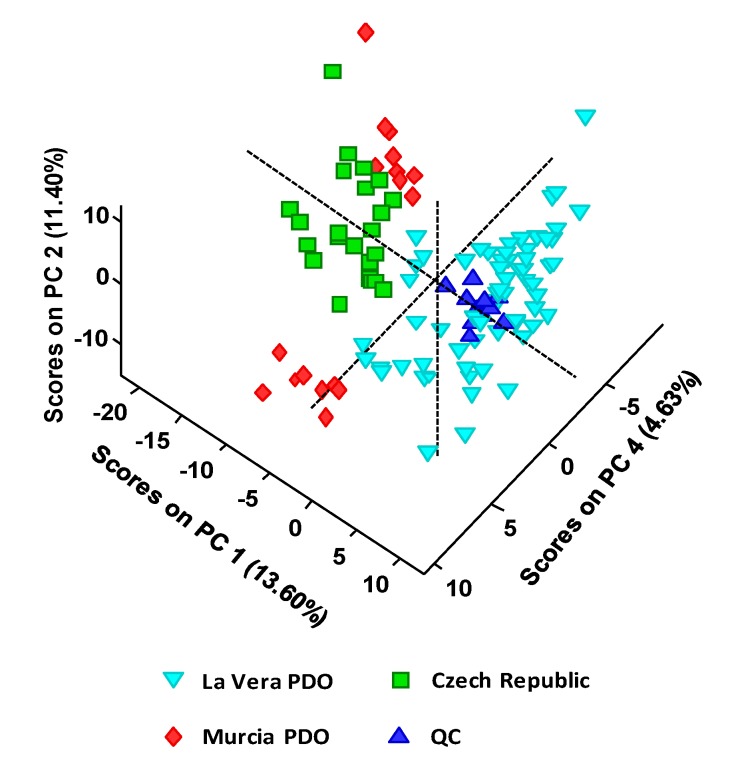
PCA 3D score plot of PC1 vs. PC2 vs. PC4 when UHPLC-HRMS fingerprints were employed as chemical descriptors for the classification of the analyzed paprika samples according to their PDO and production region.

As a first approach, a non-supervised exploratory PCA was performed with the obtained UHPLC-HRMS metabolomics fingerprints. For that purpose, sample UHPLC-HRMS raw data were first processed with MSConvert software to obtain an Excel file with the profile of peak intensities as a function of *m/z* values and retention times for all the chemical features detected. Obviously, the amount of ions registered that way is huge. Therefore, to reduce the data complexity and simplify the chemometric treatment, a threshold peak filter of an absolute intensity of 10^5^ was applied, removing all the chemical features below this value (most of them considered to be chemical noise). Data was further processed with R software to obtain the final data matrix including the non-targeted UHPLC-HRMS metabolomics fingerprints of the 105 analyzed paprika samples and the QCs. This data matrix was then submitted to PCA. Figure 2 shows the obtained PCA 3D score plot of PC1 vs. PC2 vs. PC4 for the classification of the analyzed paprika samples.

As can be seen, QCs are well clustered, revealing the good performance of the proposed UHPLC-HRMS methodology as well as the feasibility of the obtained chemometric results. As expected, QCs are clustered in the same area of La Vera PDO samples due to the higher contribution of this group of samples to the QC composition (see QC preparation in Material and Methods section). Regarding the obtained sample distribution by PCA, La Vera PDO samples are completely separated from the other two groups of samples, exhibiting positive scores on PC1. Discrimination is also more or less observed for the other two groups of samples. Besides, Murcia PDO samples were also separated into two groups by PCA, according to their two taste varieties (sweet and spicy). 

### 3.3. Sample Classification by PLS-DA

**Figure 3:**
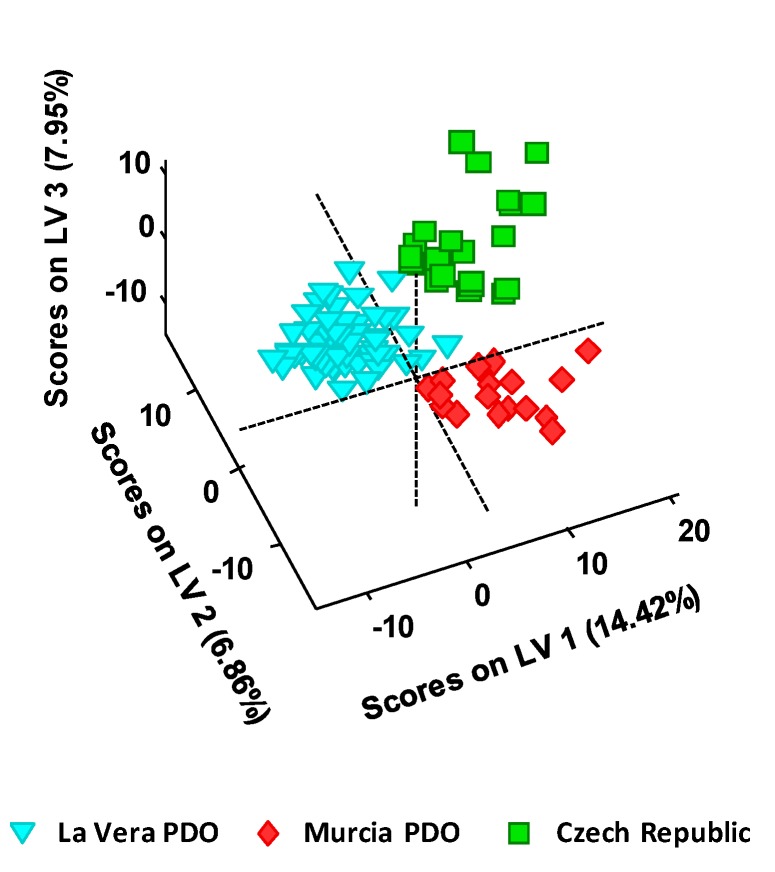
PLS-DA 3D score plot of LV1 vs. LV2 vs. LV3 when UHPLC-HRMS fingerprints were employed as chemical descriptors for the classification of the analyzed paprika samples according to their PDO and production region.

The obtained UHPLC-HRMS fingerprints were also submitted to PLS-DA to perform a supervised sample classification. For that purpose, the X-data matrix was the same as the one used for the PCA, without including QCs, and the Y-data matrix included the membership of each paprika sample. In this case, a total of four LVs were required for the optimal PLS-DA model. The obtained PLS-DA 3D score plot of LV1 vs. LV2 vs. LV3 is depicted in Figure 3.

As expected, results considerably improved when employing the classificatory PLS-DA in comparison to the exploratory PCA method. Not only the three groups of paprika samples are perfectly discriminated concerning their PDO and production regions, but more compacted groups are also obtained.

**Figure 4:**
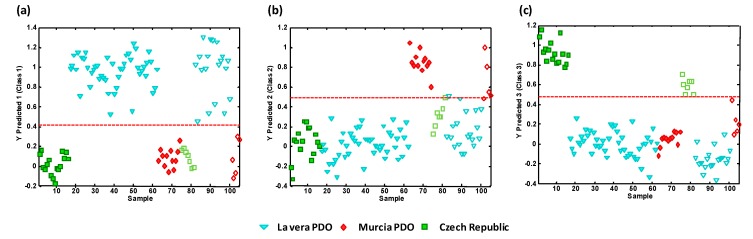
PLS-DA classification plots according to the paprika production region. (**a**) La Vera PDO vs. other classes; (**b**) Murcia PDO vs. other classes; (**c**) Czech Republic vs. other classes. Sample assignment: triangle = La Vera PDO, rhombus = Murcia PDO, square = Czech Republic. Filled symbols = calibration set, empty symbols = validation/prediction set. The dashed line means the classification boundary.

To demonstrate the applicability of the proposed UHPLC-HRMS metabolomics fingerprinting strategy for the classification and the authentication of the analyzed paprika samples according to their PDO and production regions, PLS-DA validation was performed by studying the classification rates for the PLS-DA models comparing one paprika group against the other two. For that purpose, the PLS-DA models were built by employing a calibration set composed of 70% of the samples belonging to each sample group, and then the other 30% of the samples (randomly selected) were employed as a test set for prediction purposes. Figure 4 shows the classification plots obtained for (**a**) La Vera PDO samples vs. all the other samples, (**b**) Murcia PDO samples vs. all the other samples, and (**c**) Czech Republic samples vs. all the other samples. The dashed line indicates the classification boundary, so all the samples belonging to the targeted paprika class were located to the top while samples belonging to the other two sample groups were located to the bottom. Besides, calibration samples were located to the left (with filled symbols), and those employed for prediction purposes were on the right of the plots (with empty symbols).

Excellent results were obtained with a 100% classification rate in the three PLS-DA models under study. These results demonstrate the feasibility of the proposed UHPLC-HRMS metabolomics fingerprinting strategy to obtain good sample chemical descriptors to authenticate the PDO and production region of paprika samples.

### 3.4. Sample Classification by PLS-DA According to Taste Varieties

**Figure 5:**
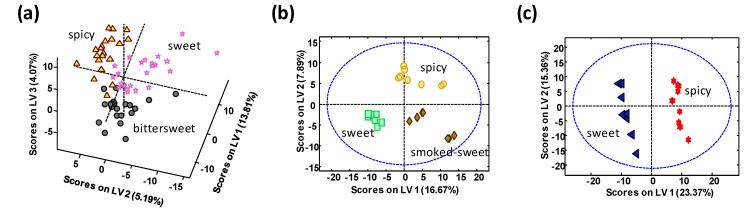
PLS-DA score plots when using UHPLC-HRMS metabolomics fingerprints as chemical descriptors for the classification of (**a**) La Vera PDO, (**b**) Murcia PDO, and (**c**) Czech Republic paprika samples according to their different taste varieties.

UHPLC-HRMS metabolomics fingerprints were also evaluated as sample chemical descriptors to address sample classification according to the different taste varieties for each paprika PDO or production region, and the best PLS-DA models obtained are represented in Figure 5.

As can be seen, the proposed methodology is also able to discriminate among the different taste varieties for each paprika PDO or production region. In the case of La Vera PDO paprika (Figure 5a), bittersweet samples tend to be located depicting negative LV1 and LV2 values, while sweet paprika variety tends to be located with negative LV2 but positive LV1 values. LV3 seems to be responsible for the discrimination of the spicy taste. Although variety discrimination is not 100% perfect, these results are very acceptable taking into account that all these samples are produced in a very small region, in comparison to the other paprika groups analyzed. For the cases of Murcia PDO paprika (Figure 5b) and Czech Republic paprika (Figure 5c), perfect discrimination among their different flavor varieties was accomplished.

## 4. Conclusions

In the present work, the feasibility of non-targeted UHPLC-HRMS (Orbitrap) fingerprints as appropriate sample chemical descriptors for the characterization, classification, and authentication of paprika samples according to both their PDO and production region and their different taste varieties has been demonstrated.

The proposed characterization and classification method has the advantage that the identification of specific metabolite compounds is not required to deal with sample authentication, as non-targeted fingerprints based on HRMS signal intensities as a function of *m/z* values and retention times are treated by the chemometric approaches.

Unsupervised exploratory analysis performed by PCA and supervised classification carried out by PLS-DA by using the obtained non-targeted UHPLC-HRMS fingerprints showed excellent discrimination capabilities of the different paprika production regions under study (La Vera PDO, Murcia PDO, and the Czech Republic paprika). PLS-DA model validations resulted in 100% classification rates in both calibration and prediction steps.

Besides, the proposed methodology also exhibited perfect discrimination and authentication capabilities among the different paprika taste varieties of each of the production regions studied—even in the case of La Vera PDO samples, where three different taste varieties (sweet, bittersweet, and spicy) are produced within a small geographical area in comparison to Murcia PDO and the Czech Republic samples.
